# Systemic immune changes accompany combination treatment with immunotoxin LMB‐100 and nab‐paclitaxel

**DOI:** 10.1002/cam4.5290

**Published:** 2022-10-08

**Authors:** Guillaume Joe Pegna, Min‐Jung Lee, Cody J. Peer, Mehwish I. Ahmad, David J. Venzon, Yunkai Yu, Akira Yuno, Seth M. Steinberg, Liang Cao, William D. Figg, Renee N. Donahue, Raffit Hassan, Ira Pastan, Jane B. Trepel, Christine Alewine

**Affiliations:** ^1^ Laboratory of Molecular Biology National Cancer Institute, National Institutes of Health Bethesda Maryland USA; ^2^ Medical Oncology Program National Cancer Institute, National Institutes of Health Bethesda Maryland USA; ^3^ Developmental Therapeutics Branch National Cancer Institute, National Institutes of Health Bethesda Maryland USA; ^4^ Clinical Pharmacology Program National Cancer Institute, National Institutes of Health Bethesda Maryland USA; ^5^ Office of Research Nursing National Cancer Institute, National Institutes of Health Bethesda Maryland USA; ^6^ Biostatistics and Data Management Section National Cancer Institute, National Institutes of Health Bethesda Maryland USA; ^7^ Genetics Branch National Cancer Institute, National Institutes of Health Bethesda Maryland USA; ^8^ Laboratory of Tumor Immunology and Biology National Cancer Institute, National Institutes of Health Bethesda Maryland USA; ^9^ Thoracic and Gastrointestinal Malignancies Branch, Center for Cancer Research National Cancer Institute, National Institutes of Health Bethesda Maryland USA; ^10^ Knight Cancer Institute Oregon Health & Science University Portland Oregon USA; ^11^ Astra Zeneca Gaithersburg Maryland USA; ^12^ Oral and Maxillofacial Surgery Kumamoto University Hospital Kumamoto Japan

**Keywords:** capillary leak syndrome, immunotherapy, immunotoxins, LMB‐100, mesothelin, pancreatic cancer

## Abstract

LMB‐100 is a novel immune‐conjugate (immunotoxin) that targets mesothelin. A phase 1/2 clinical trial was conducted (NCT02810418) with primary objectives assessing the safety and efficacy of LMB‐100 ± nab‐paclitaxel. Participant blood samples were analyzed for changes in serum cytokines and circulating immune cell subsets associated with response or toxicity. On Arm A, participants (*n* = 20) received standard 30‐minute LMB‐100 infusion with nab‐paclitaxel. Although clinical efficacy was observed, the combination caused intolerable capillary leak syndrome (CLS), a major toxicity of unclear etiology that affects many immunotoxin drugs. Participants developing CLS experienced rapid elevations in IFNγ and IL‐8 compared to those without significant CLS, along with midcycle increases in Ki‐67‐ CD4 T cells that were CD38, HLA‐DR, or TIM3 positive. Additionally, a strong increase in activated CD4 and CD8 T cells and a concurrent decrease in Tregs were seen in the single Arm A patient achieving a partial response. In Arm B, administration of single agent LMB‐100 to participants (*n* = 20) as a long infusion given over 24–48 h was investigated based on pre‐clinical data that this format could reduce CLS. An optimal dose and schedule of long infusion LMB‐100 were identified, but no clinical efficacy was observed even in patients receiving LMB‐100 in combination with nab‐paclitaxel. Despite this, both Arm A and B participants experienced increases in specific subsets of proliferating CD4 and CD8 T cells following Cycle 1 treatment. In summary, LMB‐100 treatment causes systemic immune activation. Inflammatory and immune changes that accompany drug associated CLS were characterized for the first time.

## Graphical abstract

LMB‐100 is an immunotoxin directed against mesothelin, a surface protein expressed by over 85% of pancreatic adenocarcinomas as well as an estimated 30% of all solid tumors. Immunotoxin therapies, while potentially promising, are limited by their side effect profiles, most notably capillary leak syndrome (CLS). This study provides the most detailed assessment to date of the systemic immune and inflammatory changes occurring in patients following LMB‐100 treatment and identifies those specific to patients who develop CLS. These findings illuminate the systemic effects of immunotoxin treatment in patients and could ultimately lay the groundwork for new combinations capable of harnessing the immune activating effects of immunotoxin therapy while avoiding dangerous toxicities such as CLS.

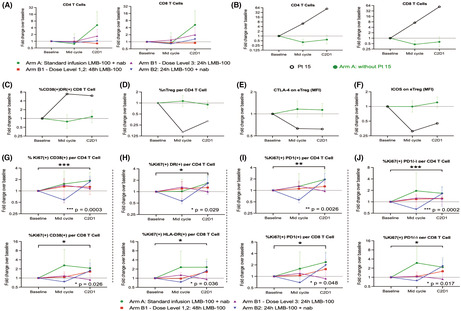

## 1 INTRODUCTION

Pancreatic cancer is a highly aggressive malignancy associated with a 5‐year overall survival rate of ~10%. At diagnosis, most patients will have regionally advanced or metastatic disease.^1^ More than 90% of pancreatic cancer cases are histologically classified as pancreatic ductal adenocarcinomas (PDAC) while the remainder include neuroendocrine and diverse exocrine pancreatic cancers.^2^ Prognosis remains poor and pancreatic cancer is currently the third leading cause of cancer death.^1^, ^3^ For metastatic or unresectable PDAC, multiagent chemotherapy is the mainstay of therapy, with FOLFIRINOX demonstrating the greatest median overall survival (OS) of 11.1 months.^4^, ^5^, ^6^ A dismal median OS of under six months is seen following second‐line chemotherapy regimens.^7^ Targeted agents may be considered in a minority of patients with actionable somatic or germline mutations as well as those with microsatellite instability‐high or tumor mutational burden‐high tumors.^8^, ^9^, ^10^, ^11^

LMB‐100 (previously called RG7787 and Ro6927005) is a second‐generation immunotoxin(iTox) targeting cell surface bound mesothelin, found on greater than 85% of PDAC.^12^ LMB‐100 is composed of a humanized anti‐mesothelin Fab linked to a recombinantly modified Pseudomonas exotoxin A (PE). Upon endocytosis by the targeted cells, PE is released into the cytoplasm resulting in inactivation of elongation factor‐2 and subsequent cell death.^13^, ^14^, ^15^ Phase 1 studies of LMB‐100 identified a single agent maximum tolerated dose (MTD) of 140 mg/kg and the dose‐limiting toxicity (DLT) of capillary leak syndrome (CLS).^16^ The combination of LMB‐100 with nab‐paclitaxel in patients with PDAC was evaluated in a phase 1/2 clinical trial based upon preclinical evidence of synergy that resulted in complete and durable anti‐tumor responses.^17^ Clinical efficacy of the combination was observed in PDAC patients both naïve and previously treated with nab‐paclitaxel and exclusively in participants with MSLN expression in ≥40% of cancer cells in archival tumor tissue, suggesting a contribution from LMB‐100. However, increased incidence of CLS made the combination regimen difficult to tolerate.^18^

Preclinical studies and pharmacokinetic modeling taking into account the ~1 h half‐life of LMB‐100 have suggested that administering prolonged infusions of LMB‐100 may maintain anti‐tumor efficacy despite decreasing peak plasma concentration (*C*_max_).^19^, ^20^ We hypothesized that decreasing *C*_max_ by administering LMB‐100 as a long infusion would result in decreased incidence of non‐specific, off‐target toxicities like CLS without compromising clinical efficacy. To evaluate this, we conducted a clinical trial to assess the safety and tolerability of long infusion LMB‐100 with or without nab‐paclitaxel. Correlative studies were performed to identify systemic immune changes that accompany LMB‐100 administration.

## 2 PATIENTS AND METHODS

### 2.1 Study design and treatment

**FIGURE 1:**
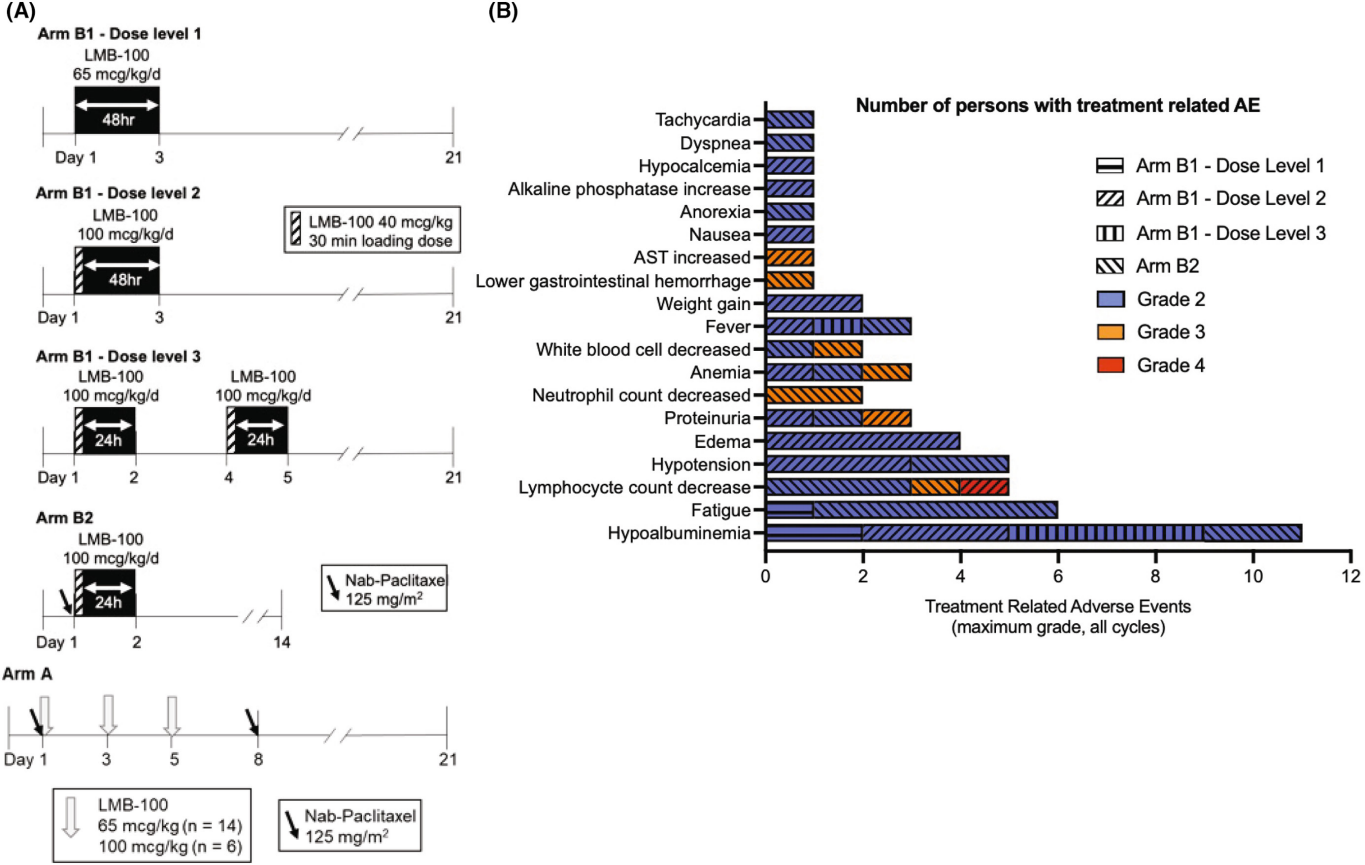
Arm B dosing regimens and Adverse Events. (A) Dosing schema. (B) All AEs ≥ grade 2 attributable to LMB‐100 with maximum grade reported by each participant recorded

This open‐label, phase I study was conducted at the NCI Center for Cancer Research (Bethesda, MD; NCT02810418). The study was comprised of two arms: Arm A in which patients received standard format LMB‐100 (30‐minute infusion on Days 1, 3 and 5 of each 21‐day cycle) with standard nab‐paclitaxel (125 mg/m^2^ on days 1 and 8) and Arm B in which patients received long infusion LMB‐100 (24‐ or 48‐h infusion) as a single agent (Arm B1) or with nab‐paclitaxel (Arm B2). Arm A clinical and limited correlative data was previously reported.^18^ The primary endpoint for Arm B1 was to identify the optimal dose and schedule of LMB‐100 when given as a long infusion. The primary objective of Arm B2 was to evaluate the safety and tolerability of the optimally dosed long infusion LMB‐100 when given in combination with nab‐paclitaxel. Secondary endpoints included categorization of adverse events (AEs), pharmacokinetic (PK) assessment, frequency of anti‐drug antibody (ADA) formation, assessment of serum tumor marker CA 19‐9 and objective radiographic response. Arm B1 utilized a modified 3 + 3 design with a flexible dose escalation scheme. Patients were accrued to 3 dose levels (DLs) with schedules as defined in Figure 1A. Choice of initial dose was based on modeling estimates to target a steady‐state plasma concentration of at least 50 ng/ml (Figure S1A). Dose and schedule were as per Figure 1A. Patients on Arm B1 received up to 2 cycles (21 days/cycle) of treatment. For Arm B2, participants received nab‐paclitaxel (125 mg/m^2^ on day 1) with long infusion LMB‐100 for a maximum of 3 cycles (14 days/cycle). Duration of treatment was limited to 2–3 cycles since previous studies have shown that most participants develop neutralizing ADAs following repetitive dosing.^16^ Nab‐paclitaxel preceded LMB‐100 administration by 30 min based upon preclinical murine studies demonstrating increased toxicity if LMB‐100 was given first. All patients received acetaminophen, diphenhydramine, and ranitidine premedication prior to LMB‐100. Ondansetron was available as needed for nausea. Dose reductions of nab‐paclitaxel were permitted as per package insert. Nab‐paclitaxel used in this study was obtained from commercial sources. LMB‐100 was manufactured by Roche then transferred to NCI.

### 2.2 Patients

Persons ≥18 years old with advanced or recurrent histologically confirmed mesothelin‐expressing (>20% of cells positive on archival sample) solid tumor malignancy previously treated with at least one line of standard chemotherapy were eligible for Arm B1. Histologically confirmed PDAC was required for enrollment on Arm B2 and prior nab‐paclitaxel was not permitted within 4 months of study enrollment. Other requirements included: measurable disease per RECIST version 1.1, Eastern Cooperative Oncology Group performance status 0–2, adequate organ function including baseline documentation of left ventricular ejection fraction ≥50% by echocardiogram, and ambulatory oxygen saturation >88%. See full Eligibility Criteria in Supplementary Methods for complete requirements. The study was conducted in accordance with FDA regulations and Good Clinical Practice guidelines. The study protocol was approved by the NCI Institutional Review Board and written informed consent was obtained from all patients participating.

### 2.3 Clinical assessments

Pre‐specified severe hematologic toxicities and most grade ≥3 nonhematologic toxicities (excluding clinically insignificant electrolyte abnormalities) were considered dose‐limiting toxicities (DLTs) if at least possibly related to LMB‐100 and occurring within 21 days of initial LMB‐100 administration. Common Terminology Criteria for Adverse Events (CTCAE) version 4.0. was used to grade adverse events. Tumor response was assessed per RECIST version 1.1 based on changes between imaging studies performed at baseline and following completion of the 6‐week treatment course. CA 19‐9 was measured at baseline, the start of each cycle, and end of treatment.

### 2.4 Pharmacokinetic analyses

LMB‐100 concentrations in patient plasma was measured by validated ELISA through contract with Frederick National Laboratory for Cancer Research operated by Leidos Biomedical Research, Inc. as described previously.^18^ Pharmacokinetic parameters were estimated using noncompartmental methods. Pharmacokinetic analyses were performed using Phoenix WinNonlin v8.1 (Certara) as per FDA 21CFR11 guidance. GraphPad Prism v8 was used for all plots and statistical tests.

### 2.5 ADA analysis

Patient ADAs were assessed using a validated screening ELISA as we have described previously.^18^ Testing was performed by contract with Frederick National Laboratory for Cancer Research operated by Leidos Biomedical Research, Inc.

### 2.6 Peripheral cytokines analysis

Blood samples were collected in BD vacutainer serum tubes, separated within 4 h, and stored in aliquots at −80°C until use. The samples were tested for multiple cytokines, using clinically validated custom V‐PLEX assay plates on an electrochemiluminescence platform, according to manufacturer's instructions (Meso Scale Discovery).

### 2.7 Immune subset analysis

Peripheral blood samples were collected in Cell Preparation Tubes™ with sodium citrate (BD Vacutainer CPT Tubes, BD Biosciences, San Jose, CA). Peripheral blood mononuclear cells (PBMCs) were obtained by centrifugation and viably frozen until analysis. Multiparameter flow cytometric analysis was performed on PBMCs as described previously.^21^, ^22^ See Supplementary Methods for further details about immune subset analysis.

### 2.8 Statistical analysis

Statistical analyses were performed using SAS/ STAT software (SAS Institute Inc) versions 14.1 (S.M.S.) and 14.3 (D.J.V.) or in GraphPad Prism (v7.01, or 8). Graphs were generated using Microsoft Excel or GraphPad Prism. Statistical tests used for results assessment are described individually in the figure legends. Analysis of variance (ANOVA) was applied when data were consistent with distributional assumptions.

## 3 RESULTS

### 3.1 Arm B patient population

Twenty patients were enrolled to Arm B between June 2017 and October 2018. Fifteen were treated with escalating doses of single‐agent long infusion LMB‐100 (Arm B1): 3 at dose level 1 (DL1) and 6 each on dose levels 2 (DL2) and 3 (DL3). Five patients received long infusion LMB‐100 with nab‐paclitaxel on Arm B2. Dosing schemes are shown in Figure 1A. Patients had received a median of 3 prior systemic treatments. Other patient demographics are listed in Table 1.

**TABLE 1:** Baseline patient demographics and clinical characteristics

Characteristics	Value (% or range)
No. of patients	20
Arm/Dose level (if applicable)	
B1/Dose level 1	3 (15%)
B1/Dose level 2	6 (30%)
B1/Dose level 3	6 (30%)
B2	5 (25%)
Tumor type	
Pancreatic adenocarcinoma	17 (85%)
Ampullary	1 (5%)
Mesothelioma	1 (5%)
Colorectal	1 (5%)
Median age – year	60 (34–78)
Gender	
Male	12 (60%)
Female	8 (40%)
ECOG PS 0–1	19 (95%)
Prior therapies	
Surgery	9 (45%)
Radiation	12 (60%)
Median no. systemic treatments	3 (1–6)
Sites of disease	
Liver	11 (55%)
Lung	12 (60%)
Other	14 (70%)
Ascites	5 (25%)
Median CA19‐9^a^	836 (13.2–94,210)

^a^ pancreatic adenocarcinoma patients only.

### 3.2 Arm B safety and tolerability

Twelve patients completed the full course of study treatment. Severe AEs (grade 3 or greater) unrelated to treatment are reported in Table S1. Treatment‐related AEs (TRAEs) ≥grade 2 are shown in Figure 1B. The most common TRAEs across all Arm B cohorts were hypoalbuminemia (55%), fatigue (30%), lymphocyte count decrease (25%), hypotension (25%), and edema (20%). All TRAEs were fully reversible and resolved before the start of the next cycle, except for fatigue reported by Arm B2 participants. Time course of hypoalbuminemia and edema, both associated with CLS, were as described previously for Arm A patients.^18^ Patients on DL1 of Arm B1 experienced minimal drug‐related toxicity. Significant edema from CLS related to LMB‐100 (defined as weight gain ≥5 kg) occurred in 3 of 6 patients receiving DL2, and 1 of these DL2 patients also experienced DLT of grade 3 proteinuria. The patient with proteinuria was discontinued from study treatment after Cycle 1 and proteinuria resolved spontaneously. Arm B1 DL3 was well tolerated without evidence of CLS. One patient receiving DL3 experienced an investigator specified DLT for increased creatinine (grade 1) that resolved with hydration, but delayed study drug administration. A single‐dose adaptation of DL3 was chosen for exploration in combination with nab‐paclitaxel in Arm B2. Grade 3 TRAEs of neutrophil count decrease (*n* = 2), lymphocyte count decrease (*n* = 1), anemia (*n* = 1), and lower gastrointestinal hemorrhage (*n* = 1), all attributed to nab‐paclitaxel, were observed. One patient experienced toxicites of grade 2 hypotension, sinus tachycardia and fatigue lasting for 10–12 days during Cycle 1. Clinical work‐up, including echocardiogram, was unrevealing of an etiology and this patient was removed from further treatment. All other Arm B2 TRAEs >grade 2 corresponded with known toxicities of nab‐paclitaxel and no CLS was seen in this arm.

### 3.3 Arm B PK analysis

**FIGURE 2:**
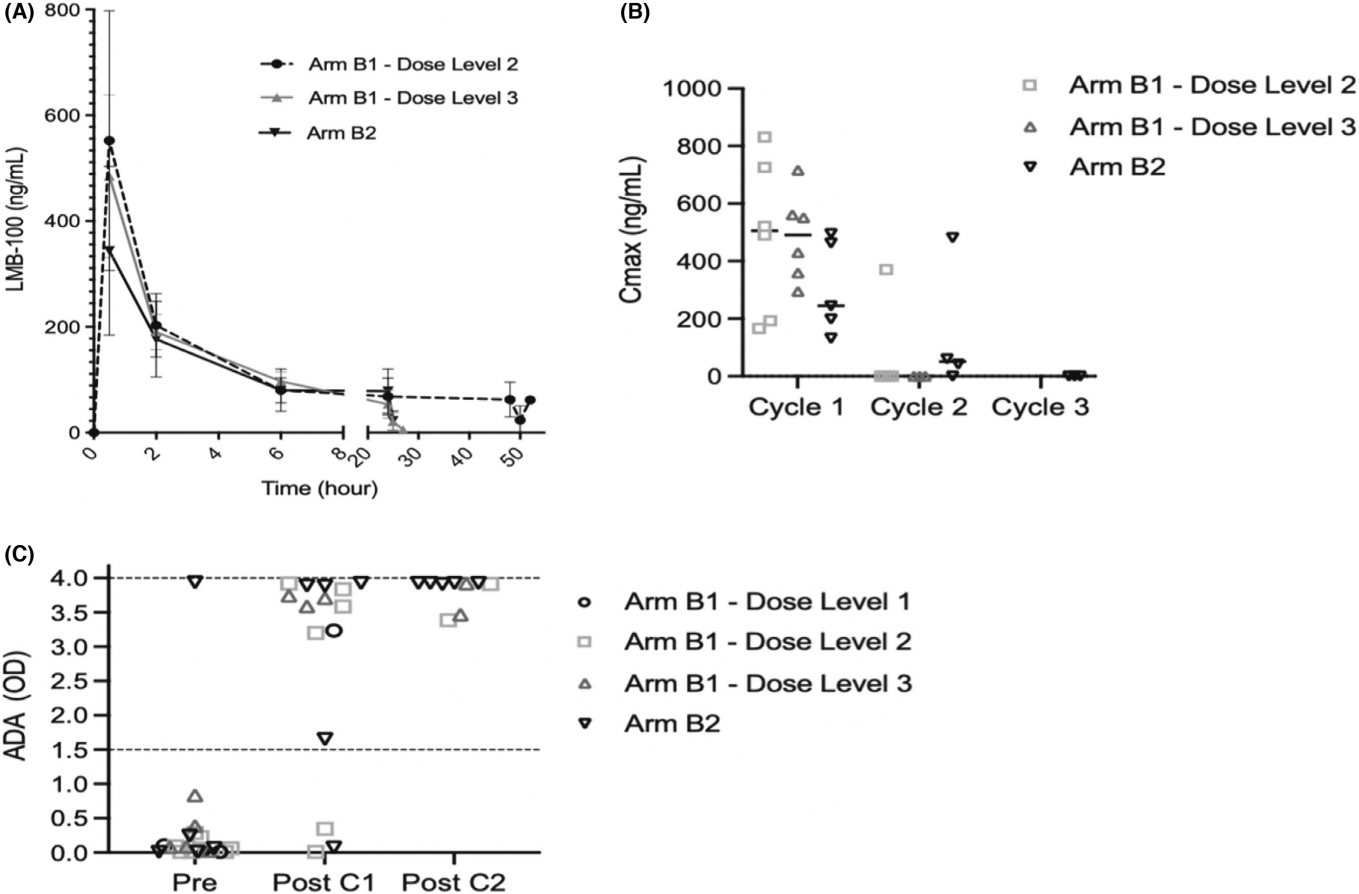
Long infusion LMB‐100 pharmacokinetics and ADA formation. (A) LMB‐100 plasma concentrations during C1D1 over time, plotted as mean LMB‐100 concentration ±SD. The 0 timepoint represents start of pre‐load infusion. (B) LMB‐100 *C*_max_ by patient and cycle, solid bar indicates median. (C) ADA measurements for each patient drawn pretreatment (Pre), at end of cycle 1 (Post C1), and end of cycle 2 (Post C2). ADA level higher than optical density (OD) 1.5 as marked by dashed line predicts drug *C*_max_ below estimated efficacy threshold. OD 4.0 is maximal measurement of the assay

Cycle 1 PK data was available for all patients treated on Arm B. Highly erratic *C*_max_ and steady state (*C*_ss_) plasma LMB‐100 concentrations were observed in the 3 patients treated with 65 mcg/kg/day on DL1, resulting in mean AUC of 2670 hr ng/ml with standard deviation (±2778 h ng/ml) nearly equal to the mean (Table 2; Figure S2B). The study team determined that the technical capability to deliver the planned drug volume exceeded the specifications of available continuous venous infusion pumps and an amendment was made to the study protocol to limit lowest dosing to 100 mcg/kg/day. Further, low‐level pre‐existing ADAs present in patient plasma might bind and sink a small amount of drug trickling into the circulation, limiting drug accessibility to target tumor tissue. For this reason, a 40 mcg/kg loading dose administered over 30 min was instituted (Figure 1A). More consistent PK profiles were observed for DL2, DL3 and Arm B2 (Table 2). In these patients, a rapid spike in plasma drug concentration occurred with the loading dose, which tailed out by 6 h to steady‐state plateau concentrations expected with long infusion (Figure 2A). As expected, serum concentrations of LMB‐100 rapidly diminished at the end of infusion, consistent with the previously measured half‐life of ~1 h.^16^

**TABLE 2:** Pharmacokinetic summary for long infusion LMB‐100

	48 h continuous infusion		24 h continuous infusion^a^	
	Arm B1 DL1 (*n* = 3)	Arm B1 DL2 (*n* = 6)	Arm B1 DL3 (*n* = 6)	Arm B2 (*n* = 5)
*C*_max_ (ng/ml)	1124 (1892)	487 (270)	485 (154)	307 (163)
*C*_ss_ (ng/ml)	15.3 (23.7)	70.2 (28.2)	75.4 (16.9)	79.4 (32.1)
AUC_last_ (h ng/ml)	2670 (2778)	4148 (1746)	2508 (388)	2417 (1006)

*Note*: Data presented as mean (SD).

Abbreviations: AUC_last_, area under the concentration‐time curve from time zero to time of last measurable concentration; DL, dose level; *C*_max_, maximum concentration; *C*_ss_, concentration of drug at steady state.

^a^ Twenty‐four hour continuous infusion pharmacokinetic parameters measured for drug administration through days 1–2 of therapy and do not include measurements from second dose given on days 4–5 of each cycle.

### 3.4 Relationship of ADA formation and LMB‐100 *C*_max_

As seen previously with standard infusion LMB‐100, median *C*_max_ for long infusion LMB‐100 declined with repeated treatment cycles. While Cycle 1 *C*_max_ exceeded 100 ng/ml in all 17 patients receiving the loading dose, only 2 of 13 patients exceeded this threshold during Cycle 2 administration (Figure 2B). Modification to a shorter 14‐day cycle length on Arm B2 to potentially deliver the second cycle before ADA development did not alleviate this problem: *C*_max_ levels exceeding 100 ng/ml during Cycle 2 administration were observed in only 1 of 4 patients. In total, only 1 of 20 patients treated on Arm B of the study had clinically meaningful, high‐titer ADA levels (defined as mean assay signal ≥1.5) prior to treatment, however, high ADA levels developed in 12 of 15 evaluable patients after Cycle 1 (Figure 2C). We have previously shown that higher ADA measurements are associated with poor to absent peak plasma concentrations of LMB‐100.^16^, ^18^ Here, we observed that higher baseline ADA measurement (mean assay signal ≥0.1) was associated with early development of high‐titer ADAs and poor C2 drug levels regardless of infusion format: 7 of 8 participants in Arm A and 4 of 4 in Arm B with pre‐treatment ADA ≥0.1 had poor C2 drug levels (Fisher's exact test, 2‐sided *p* = 1.00). However, the few patients on Arm B with low baseline ADA (<0.1) exhibited a weak tendency towards higher frequency of developing poor C2 drug levels (4 of 6 in Arm B versus 2 of 8 in Arm A, Fisher exact test, 2‐sided *p* = 0.2). Administration of LMB‐100 in a long infusion format appears to invoke earlier development of high‐titer ADAs that limit peak plasma drug levels.

### 3.5 Clinical response

No objective responses were observed in any patients on Arm B. Clinically significant decreases in CA 19‐9 tumor marker (>50% decline from baseline) were seen in 2 of 12 evaluable patients, both of whom received nab‐paclitaxel (Figure S2). Four patients stopped treatment early due to progression of disease or complications from their tumor and two additional patients withdrew from treatment after Cycle 1 due to worsening symptoms. Clinical anti‐tumor activity of long infusion LMB‐100 was judged to be unlikely.

### 3.6 Systemic inflammatory response to drug administration

**FIGURE 3:**
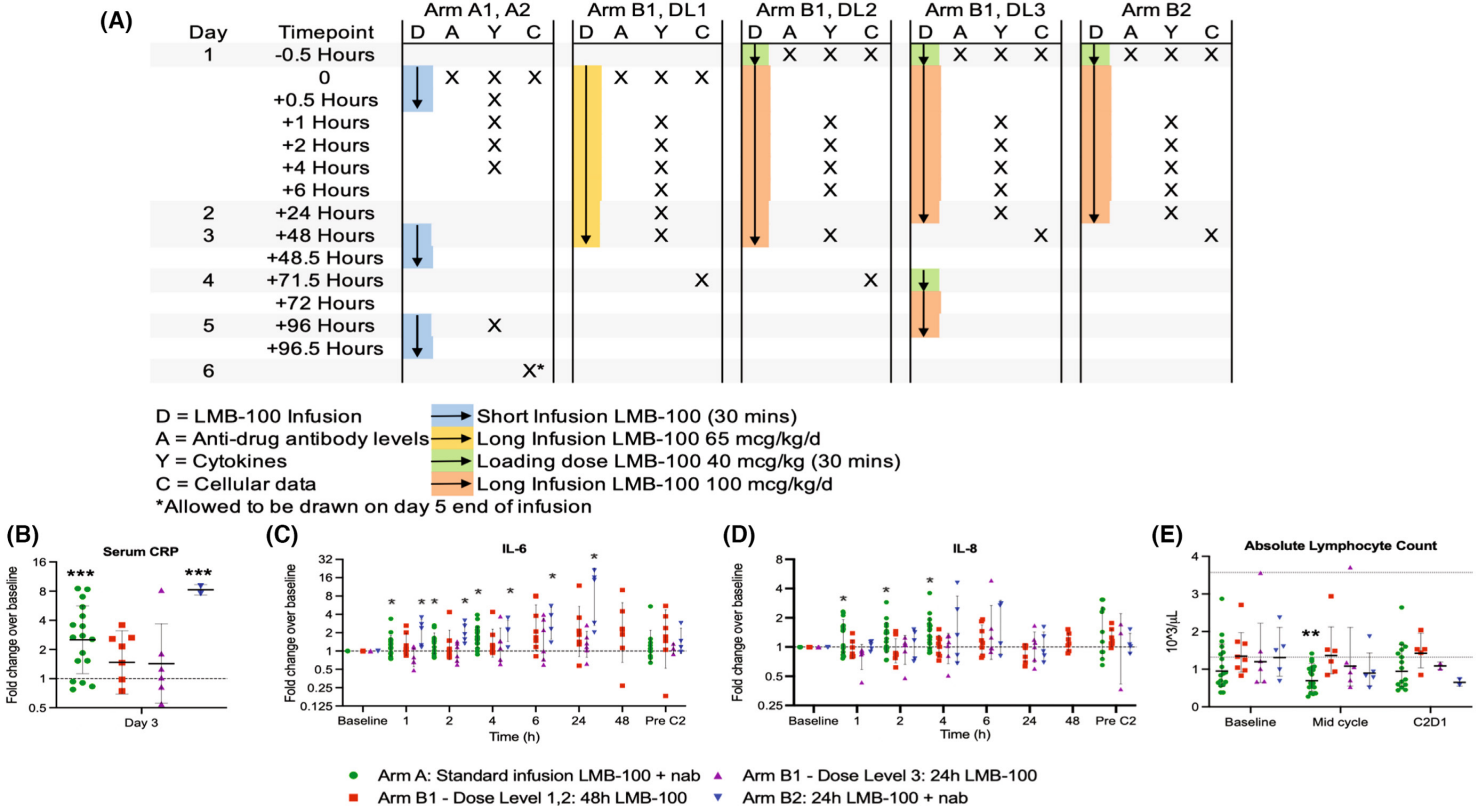
Immune changes in response to study treatment. (A) Schema depicting timepoints at which specified samples were drawn in relation to drug administration. (B) Serum CRP in treated patients, where *** indicates *p* < 0.001 for post‐hoc analysis following ANOVA of log(fold change) from mean over baseline. Horizontal bars for each group indicate geometric mean. (C, D) Serum IL‐6 and IL‐8 in treated patients where * indicates *p* < 0.05 for repeated measures ANOVA of log_10_‐transformed fold changes with Holm correction for multiple comparisons. For B–D, error bars show geometric standard deviation, and dashed line at 1 indicates pre‐treatment baseline. (E) Absolute lymphocyte counts with upper and lower limits of normal indicated by horizontal dotted lines. Statistical significance (**p < 0.01) by repeated measures ANOVA performed on log‐transformed values is indicated

To better understand the inflammatory changes caused by LMB‐100, serum C‐reactive protein (CRP) and serum cytokine concentrations, as well as circulating immune cell profiles were analyzed for patients enrolled on Arms A and B as part of pre‐planned exploratory objectives for the study. Timing of these sample collections is illustrated in Figure 3A. A median of 35 (range 19–37) patients were evaluable for CRP and cytokine analyses, depending on the analyte (Table S2). Three patients on Arm A experiencing infectious AEs during sample acquisition were excluded from the analysis to prevent confounding. We found that patients receiving standard or long‐infusion LMB‐100 with nab‐paclitaxel (Arm A, *n* = 17; Arm B2, *n* = 2) had increases in serum CRP by median fold changes >2 over baseline by study day 3 (D3) (Figure 3B). Serum CRP increase in Arm A was maintained at D5 and D8 (Figure S3). A similar trend was not observed in the Arm B1 cohorts, suggesting that co‐administration of nab‐paclitaxel is the predominant driver of CRP increase in this population.

Treatment‐related changes in plasma cytokine profiles were also evaluated. We observed no consistent changes in interferon‐gamma (IFNγ), interleukin‐1β (IL‐1β), interleukin‐4 (IL‐4), interleukin‐10 (IL‐10), interleukin‐12p (IL‐12p), interleukin‐13 (IL‐13), or TNF‐alpha (TNFα) following study treatment (Figure S4). Interleukin‐2 levels were elevated at the 4‐h time point in Arm A patients (log[fold] mean 0.24, *p* = 0.010), but not in patients on other study arms (Figure S4). As later measurements were not available in this group, the trend could not be followed further. Treatment was also associated with significant increases in serum IL‐6 in both arms receiving nab‐paclitaxel beginning at the first measurement and increasing with subsequent measurements (Arm A: log(fold) mean 0.09, *p* = 0.047, and Arm B2: log(fold) mean 0.30, *p* = 0.0029 by 1 h; increasing to Arm A log(fold) mean 0.23, *p* < 0.0001 at 4 h, and Arm B2 log(fold) mean 0.89, *p* = 0.0010 at 24 h). This IL‐6 increase was also observed in Arm B1 but did not reach statistical significance after correction for multiple comparisons (Figure 3C). A significant increase in IL‐8 was observed for Arm A patients only beginning at 1 h and continuing to the last measurement (log(fold) mean 0.15, *p* = 0.0018 at 4 h) (Figure 3D). These data suggest that nab‐paclitaxel exposure drives early increases in IL‐6 but that LMB‐100 may contribute, and that short infusion LMB‐100 stimulates IL‐8 production.

### 3.7 Changes in immune cells specific to the responding patient

**FIGURE 4:**
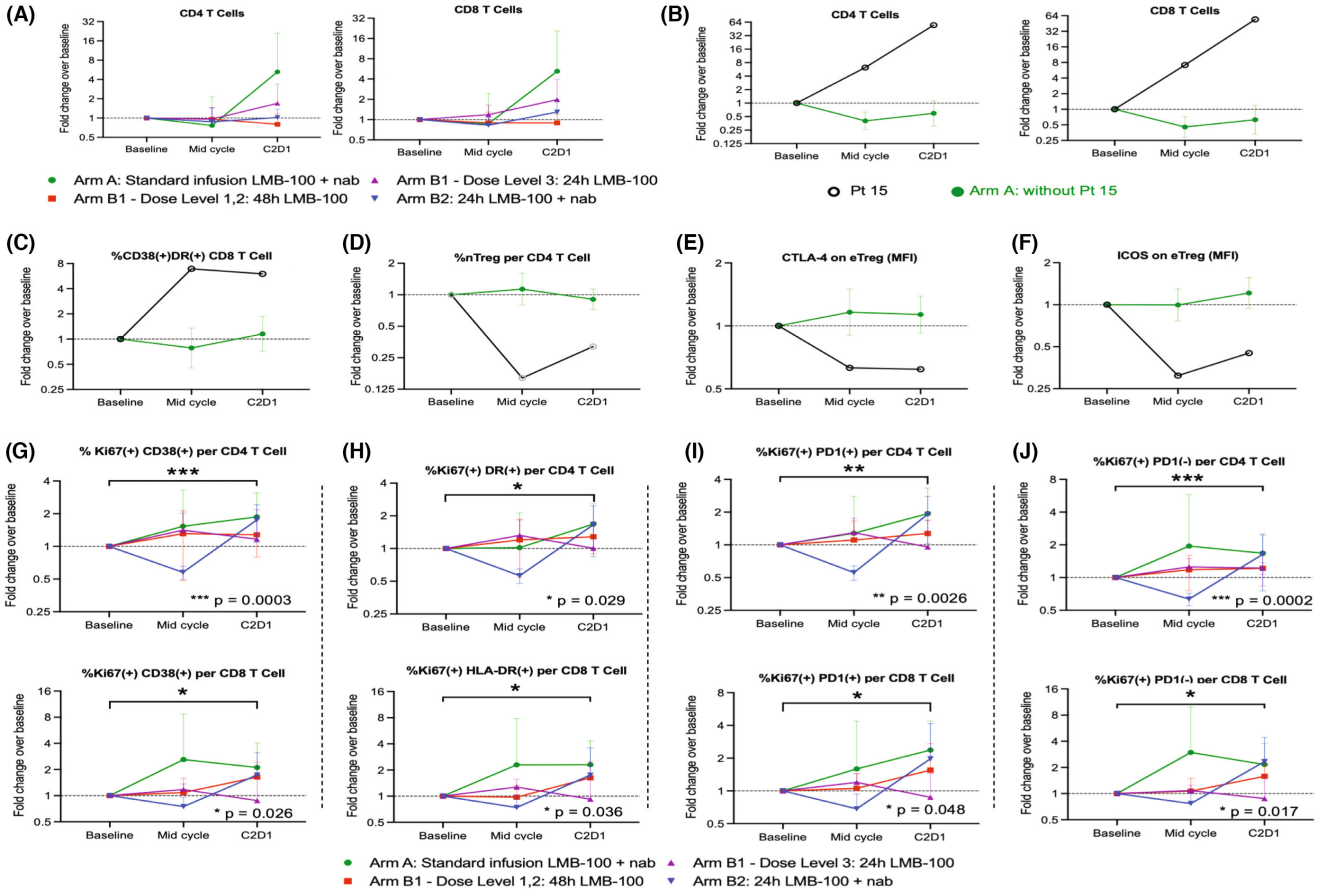
Changes in circulating immune cell subsets for the whole study population (A, G–J) or responding Patient#15 as compared to the other 15 patients in Arm A (B–F). Dashed horizontal line at 1 indicates pre‐treatment baseline. Markers indicate geometric mean and error bars show geometric standard deviation. (A) CD4 and CD8 T cell counts for all patients. (B) CD4 and CD8 T cells, (C) CD38+/DR+ CD8 T cells, (D) nTreg cells per CD4 T cells, (E) CTLA‐4 on eTreg cells, and (F) ICOS on eTreg cells with data for patient #15 broken out. (G–J) Fold change over time for indicated subsets of CD4+ and CD8+ T cells. Results are stratified by indicated study cohort on the plots, but values for the entire population were used to determine statistical significance. The *p*‐values were calculated using the exact Wilcoxon signed rank test

Circulating immune cell subsets were examined in all treated patients at baseline, 24 h after completion of infusion, and at end of cycle 1 (see schema, Figure 3A). A modest decrease in absolute lymphocyte count (ALC) was observed in Arm A patients at mid‐cycle (baseline to mid‐cycle mean log(ALC) change −0.14, *p* = 0.0058) (Figure 3E). Interestingly, there were unusual increases in total circulating CD4+ and CD8+ T cells (Figure 4A) despite the decrease in ALC within this same cohort. These increases were driven by values from a single patient (#15), the only participant in the study with an objective radiographic response to treatment. Patient#15 had large increases in both mid‐ and end‐of‐cycle CD4+ and CD8+ T cells while these subsets remained stable or trended lower in other Arm A patients (Figure 4B). In addition, more detailed analysis of CD4+ and CD8+ subsets showed this responder had increases in CD38+ DR+ CD8 T cells, a highly activated CD8 T cell subset (Figure 4C), decrease in naive Tregs (nTreg), an immunosuppressive population (Figure 4D), and decrease in CTLA‐4, and ICOS expression on effector Tregs (eTregs) (Figure 4E,F) at mid‐cycle and end of cycle as compared to others in Arm A, suggesting diminished immunosuppression. Of note, Patient #15 also had higher IFNγ levels than other patients on Arm A (Figure S4). Taken together, these data demonstrate that immune cell activation was induced by study treatment in the responding patient.

### 3.8 Changes in peripheral immune cell subsets for all treated patients

Preliminary analysis of immune cell subsets in the whole study population identified no statistically significant interarm‐ or dose level‐dependent differences (*p* > 0.10 in all outcomes by Kruskal‐Wallis test). Therefore, the impact of treatment on peripheral immune cell subsets was assessed as median fold changes across all evaluable patients (*N* = 27) compared to baseline. Overall, there was a statistically significant increase in end of cycle Ki‐67+ CD38+ and Ki‐67+ HLA‐DR+ subsets for both CD4 and CD8 T‐cells (Figure 4G,H). In addition, an overall increase in % of Ki67+ CD4 and CD8 T‐cells was observed independent of cell PD‐1 status (Figure 4I,J). These increases were driven by Arms A, B1 that received 48‐h infusion, and B2, but were not apparent in Arm B1‐DL3 patients. No other differences in subsets of CD4 T cells, CD8 T cells, dendritic cells, or Tregs were seen (Figure S5). In summary, study treatment caused increases in proliferating CD4 and CD8 T cells bearing activation markers.

### 3.9 Association of systemic inflammatory response and CLS

**FIGURE 5:**
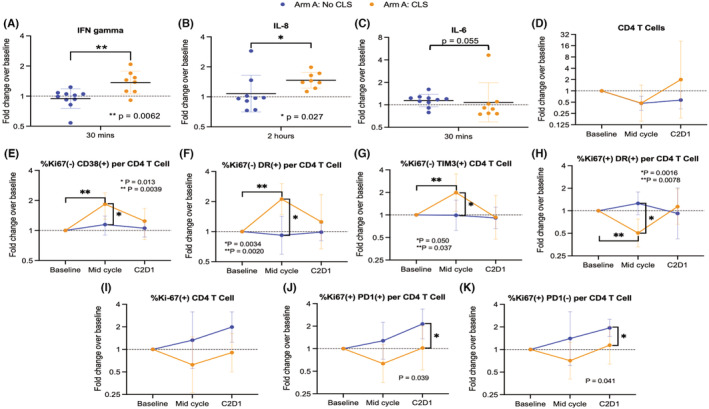
Cytokine and circulating immune cell changes associated with CLS in patients receiving standard infusion LMB‐100 with nab‐paclitaxel. Horizontal bar indicates geometric mean and error bars show geometric standard deviation. Dashed line indicates pre‐treatment baseline. For A–C, statistical significance was determined using the Wilcoxon rank sum test. For E–K, ANOVA was used to identify differences between the cohorts with and without CLS. Wilcoxon signed rank test was used to assess mid‐cycle changes in E–H

If LMB‐100‐induced CLS is caused by an inflammatory response to the iTox drug, then early changes in circulating cytokines following LMB‐100 administration would be expected in patients who go on to experience CLS. To evaluate whether CLS is associated with systemic cytokine changes immediately following LMB‐100 infusion, we stratified cytokine results by those who did or did not develop “significant” CLS, as defined previously (weight gain from edema >5 kg).^18^ Due to the low incidence of CLS in Arm B (3 of 20), the analysis was limited to Arm A patients. Within this group, serum CRP levels were similar in evaluable patients with (*n* = 8) and without (*n* = 10) significant CLS, nor were differences in IL‐2, IL‐10, IL‐12p, IL‐13 or TNFα observed (Figure S6). However, patients in the CLS cohort experienced transient increase in IFNγ concentration by 30 min post‐LMB‐100 inusion (Figure 5A; Figure S6), followed by increased IL‐8 beginning at 2 h and continuing to the last measurement at 4 h (Figure 5B; Figure S6). In addition, IL‐4 decreased at 30 min in patients without CLS, and IL‐1B concentration was lower in the limited number of patients in the CLS cohort with data available (Figure S6). While IL‐6 trended up in Arm A patients overall (Figure 3C), only a marginal difference between the cohorts with and without CLS was observed at 30 min (Figure 5C; Figure S6). Taken together, these data support an association between increased early IL‐8 and IFNγ and later development of CLSCLS was also associated with differences in specific circulating CD4 T‐cell populations measured at mid‐cycle and start of Cycle 2, despite no differences in ALC (Figure S7) or total CD4 cells between the cohorts (Figure 5D). CLS patients had increased Ki67‐CD38+, Ki67‐HLA‐DR+, and Ki67‐TIM3+ cells at mid‐cycle (Figure 5E–G), and also decreased Ki67+ DR+ cells at this time point (Figure 5H). In addition, an end of cycle increase in Ki67+ cells was observed in patients without CLS that was independent of the PD‐L1 status of these cells (Figure 5I–K). No significant differences were noted in the other circulating immune subsets analyzed (Figure S7). Changes in specific CD4 T cell subsets are associated with development of clinically significant drug‐induced CLS.

## 4 DISCUSSION

In our present study, we found that long infusion LMB‐100 with or without nab‐paclitaxel was well‐tolerated, however in contrast to short infusion LMB‐100 with nab‐paclitaxel no clinical efficacy was seen. Our correlative studies of cytokine and circulating immune cell subsets of all study patients exposed to LMB‐100 allowed for detailed analysis of inflammatory and immunologic changes occurring in response to iTox (±nab‐paclitaxel) therapy. Significantly, we observed evidence of peripheral immune activation in a patient with partial response to LMB‐100 and nab‐paclitaxel. Further, we demonstrated that LMB‐100 administration resulted in increased numbers of active circulating CD4 and CD8 T cells, and identified specific changes in serum cytokines and peripheral CD4 T cell subsets associated with CLS, the major toxicity of iTox therapies.

Prior work investigating first generation mesothelin‐targeted immunotoxin SS1P, the predecessor to LMB‐100, found that combination of immunotoxin with a lymphocyte‐depleting regimen to prevent ADA formation resulted in durable, near complete responses in mesothelioma patients after just 2 cycles of treatment.^23^ These responses were associated with large, transient increase in FDG‐avidity on PET scan, consistent with massive immune infiltration. Subsequent clinical^24^ and pre‐clinical studies^25^, ^26^ have shown that co‐administration of LMB‐100 facilitates tumor response to anti‐CTLA4 and anti‐PD1 therapies, however, specific mediators of immune response induced by LMB‐100 administration have not been identified. Standard LMB‐100 infusion in patients with malignant mesothelioma caused increases in plasma IL‐6 levels 5 days after start of treatment concordant with IL‐8 increases.^24^ These are cytokine changes which our present study suggests may begin within hours of LMB‐100 administration. More importantly, our data have identified for the first time that LMB‐100 induces perturbations in circulating immune cell populations irregardless of iTox dosing schedule or concomitant chemotherapy. Specifically, LMB‐100 administration increased the proportion of proliferating, activated CD4 and CD8 T cells following a single cycle of treatment. These data provide additional evidence that iTox administration is immune‐modulating and that this class of drugs may be useful adjuvants given in combination with immunotherapy. This theory is being actively tested in current clinical trials for patients with mesothelioma (NCT04840615) and lung cancer (NCT04027946).

Unique immune changes were identified in the single responding patient on this study. As discussed previously,^18^ this participant (enrolled on the standard infusion LMB‐100 with nab‐paclitaxel arm) had not received prior nab‐paclitaxel, had a low burden of disease and excellent performance status, and had excellent LMB‐100 *C*_max_ for both treatment cycles. Here, we have shown that he developed increases in circulating CD4 and CD8 T cells within 5 days of starting treatment, including a rapid and sustained increase in highly activated CD38+ HLA‐DR+ CD8 T cells as compared to non‐responding patients. Simultaneously, a decrease was observed in immunosuppressive immune subsets such as circulating nTregs and expression of immunosuppressive functional markers CTLA‐4 and ICOS on eTregs. None of these changes were observed in other patients receiving the same treatment regimen, even those with clinically significant CA19‐9 responses. Similar increases in CD4 and CD8 T cells have been seen in gastrointestinal cancer patients responding to treatment with combination chemotherapy and immunotherapy.^27^ Moreover, increased CD38+ HLA‐DR+ CD8 T cells have been observed in breast cancer patients achieving a pathologic complete response to neoadjuvant chemotherapy.^28^ Cell changes in these responding breast cancer patients were accompanied by a sustained increase in IFNγ, a trend that was also observed in the responding patient here. Existing data does not allow us to determine whether the changes seen are specific to response to chemotherapy alone or secondary to the combination with LMB‐100, however, our findings have identified an activated immune profile associated with response to LMB‐100/ nab‐paclitaxel and suggest that maximal clinical benefit from this treatment incorporates a robust anti‐tumor immune response.

CLS is the major dose‐limiting toxicity of iTox drugs, including LMB‐100, and occurs independent of the therapeutic target of the iTox.^16^, ^29^, ^30^ The etiology for this toxicity has been debated with some suggesting that high blood concentrations of iTox upon infusion favors non‐specific drug uptake by target‐negative endothelial cells lining blood vessels, resulting in direct iTox‐mediated killing of this bystander population.^31^ Our previous work has linked CLS to endothelial cell injury, as evidenced by a strong association between increases in apoptotic circulating endothelial cells and severity of CLS.^18^ In addition, pre‐clinical studies by some in our group have shown that iTox can specifically damage proximal tubule cells of the kidney as it transcytoses.^32^ Here, we have explored a new theory, namely, that systemic inflammation caused by LMB‐100 administration releases cytokine mediators that incite immune cells resulting in immune‐mediated vascular damage and CLS symptoms. In our study, we observed that early increases in IFNγ and IL‐8 concentrations following LMB‐100 initiation were associated with the later development of CLS. A coordinated increase in both IFNγ and IL‐8 is unexpected given the known inhibitory function of IFNγ on IL‐8 production.^33^ Importantly, the low amplitude spike in IFNγ that we observed at 30 minutes post‐treament was transient; differences in IFNγ between the patients with and without CLS had resolved by the 2 h timepoint when IL‐8 began to rise. IFNγ drives polarization of CD4+ T cells towards a Th1 phenotype through IL‐12 receptor upregulation and STAT1 signaling.^34^, ^35^, ^36^, ^37^ The potential role of interferon‐mediated CD4 T cell activation in patients with CLS was supported by midcycle increases in activated, non‐proliferating HLA‐DR+ and CD38+ CD4 T‐cells, along with non‐proliferating TIM3+ CD4 T cells. While TIM3 is typically considered a marker associated with T cell exhaustion and/or dysfunction, its expression has also been described on activated CD4 T cells.^38^, ^39^ IFNγ is also a key regulator of natural killer (NK) cells and B cells, however, we did not examine these cell populations in our study. IL‐8 is an important chemotactic factor in the innate immune response. We hypothesize that increases in IL‐8 may trigger migration of innate immune cells that subsequently damage endothelial cells to cause CLS. Our data are consistent with a prior study demonstrating that *Pseudomonas* exotoxin A can directly stimulate IL‐8 production.^40^ The rapid timescale of the observed IFNγ and IL‐8 increases makes it unlikely that direct damage caused to endothelial cells by circulating iTox happens first and subsequently results in cytokine increases. Instead, our data suggest that cytokine release must precede endothelial cell damage as numerous in vitro studies have documented that iTox‐mediated cell killing requires many hours or even days to occur.^15^, ^17^ This raises the question of whether immune activation by iTox may be the underlying primary mechanism for subsequent endothelial damage and the constellation of symptoms associated with iTox‐induced CLS.

Long infusion regimens of LMB‐100 appear to cause more rapid ADA formation than repeated administration of a standard 30‐minute infusion given on alternating days. Less than 10% of patients receiving single‐agent long infusion LMB‐100 achieved clinically significant Cycle 2 *C*_max_ as compared to ~50% receiving standard infusion LMB‐100 in prior studies.^16^, ^24^ In contrast to our present findings, members of our group have previously shown that either long or standard administration of first generation mesothelin‐targeted iTox SS1P results in similar rates of early ADA formation.^41^, ^42^ Others testing the CD22‐targeted iTox IgG‐RFB4‐SMPT‐dgA in patients with B‐cell lymphoma have also observed no difference in ADA formation between bolus and continuous infusion regimens.^41^, ^42^, ^43^ Published studies of other antibody‐based therapeutics suggest that combination with nab‐paclitaxel can reduce ADA formation.^44^, ^45^ However, we have previously shown that rates of early ADAs to LMB‐100 resulting in low cycle 2 drug levels are similar for patients receiving single‐agent standard infusion LMB‐100 alone as compared to nab‐paclitaxel combination,^16^ demonstrating nab‐paclitaxel is inadequate to suppress ADA development against LMB‐100. As elevated ADAs resulting in suboptimal drug levels remain a fundamental limitation to the potential efficacy of LMB‐100, further study into alternative drug combinations and/or dosing regimens that could prevent ADA formation is needed.

In summary, our detailed analysis of peripheral immune cells and systemic cytokines in patients receiving the LMB‐100 iTox has identified activating changes in cytokines and circulating immune cell populations that occur in most patients treated with LMB‐100, uniquely in a patient with clinical response to LMB‐100/ nab‐paclitaxel, and specifically in patients who develop iTox‐mediated CLS.

## AUTHOR CONTRIBUTIONS

Study concept: Christine Alewine, Raffit Hassan, Ira Pastan. Study design: Christine Alewine, Cody J. Peer, Seth M. Steinberg, Liang Cao, William D. Figg, Jane B. Trepel. Study oversight: Christine Alewine. Patient care: Christine Alewine, Mehwish I. Ahmad. Data acquisition: Christine Alewine, Mehwish I. Ahmad, Min‐Jung Lee, Yunkai Yu, Akira Yuno. Data analysis and interpretation of data: Guillaume Joe Pegna, Min‐Jung Lee, Cody J. Peer, Mehwish I. Ahmad, David J. Venzon, Seth M. Steinberg, Renee N. Donahue, Jane B. Trepel, Christine Alewine. Writing original manuscript: Guillaume Joe Pegna, Christine Alewine. Review and revision of manuscript: all authors.

## FUNDING INFORMATION

This research was supported by the Intramural Research Program of the NIH, National Cancer Institute, Center for Cancer Research (Project No's ZIA BC 011652, ZIC SC 006537, ZIC SC 006743).

## CONFLICT OF INTEREST

J.B.T. received research funding from Syndax, EpicentRx and AstraZeneca to her institution. C.A. receives drug support from Minneamrita and AstraZeneca for clinical studies. R.H. receives research support from Bayer AG and TCR2 Therapeutics. All other authors do not have a conflict of interest.

## ETHICS STATEMENT

This open‐label, phase I study was conducted at the NCI Center for Cancer Research (Bethesda, MD; NCT02810418). The study was conducted in accordance with FDA regulations and Good Clinical Practice guidelines. The study protocol was approved by the NCI Institutional Review Board and written informed consent was obtained from all patients participating.

## Supporting information


Figure S1

Figure S2

Figure S3

Figure S4

Figure S5

Figure S6

Figure S7

Table S1

Table S2

Appendix S1

## Data Availability

Data are available upon reasonable request.
