# Supplementary figures and images for: Systemic immune changes accompany combination treatment with immunotoxin LMB‐100 and nab‐paclitaxel
Source: Cancer Med. 2022 Oct 8;12(4):4236–49. doi: 10.1002/cam4.5290 (PMC9972172; doi:10.1002/cam4.5290)

# Supplemental Figure 1. Long infusion simulated and actual PK's

**A**

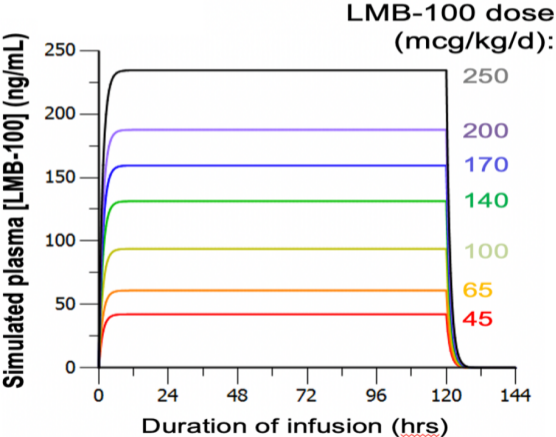

**B**

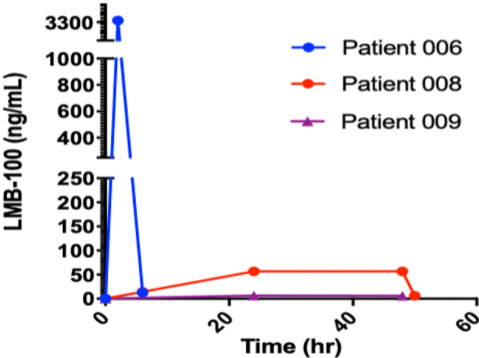

Supplement: Supplementary file 1 — Figure S1 [file CAM4-12-4236-s008.pdf]

**Supplemental Figure 2. Best CA19-9 Response**

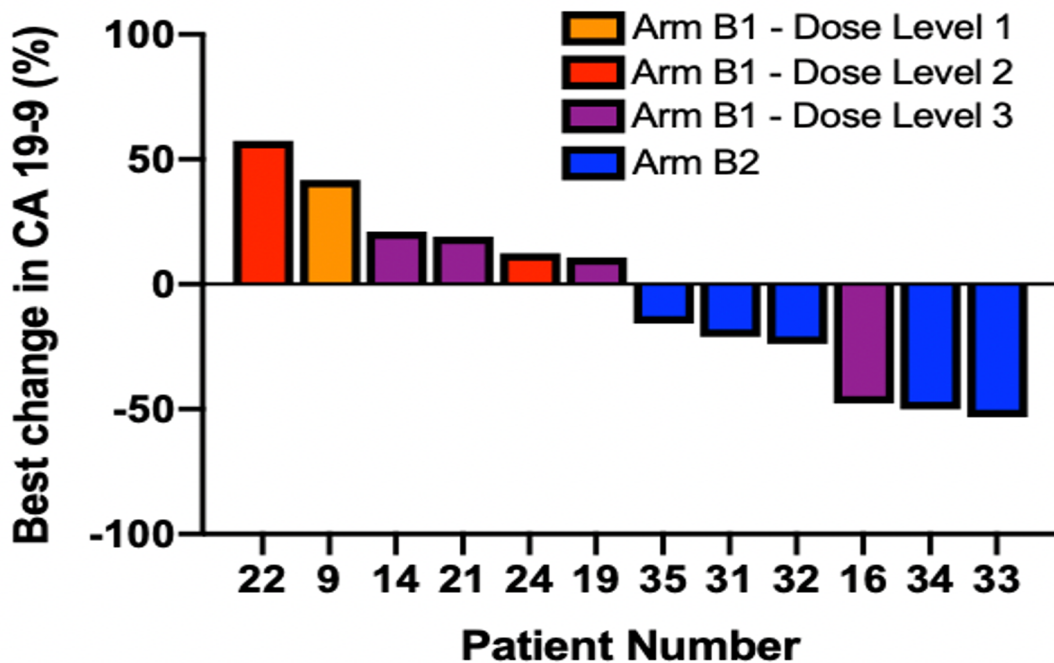

Supplement: Supplementary file 2 — Figure S2 [file CAM4-12-4236-s003.pdf]

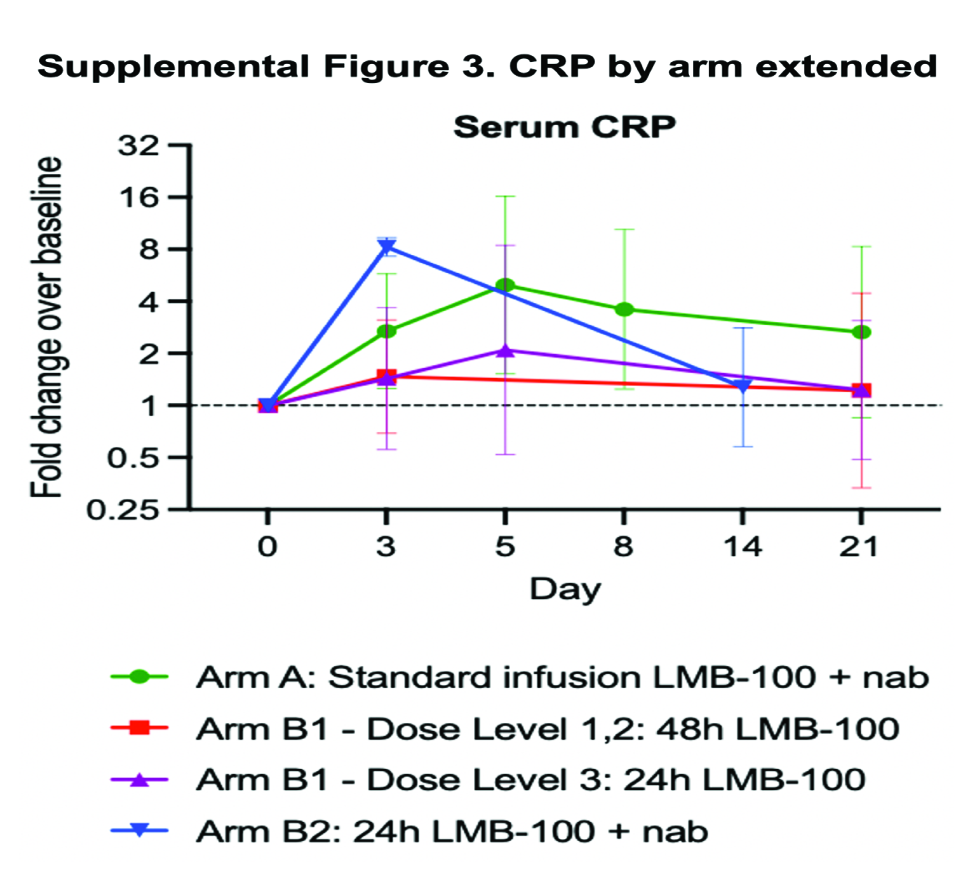

Supplement: Supplementary file 3 — Figure S3 [file CAM4-12-4236-s004.tif]

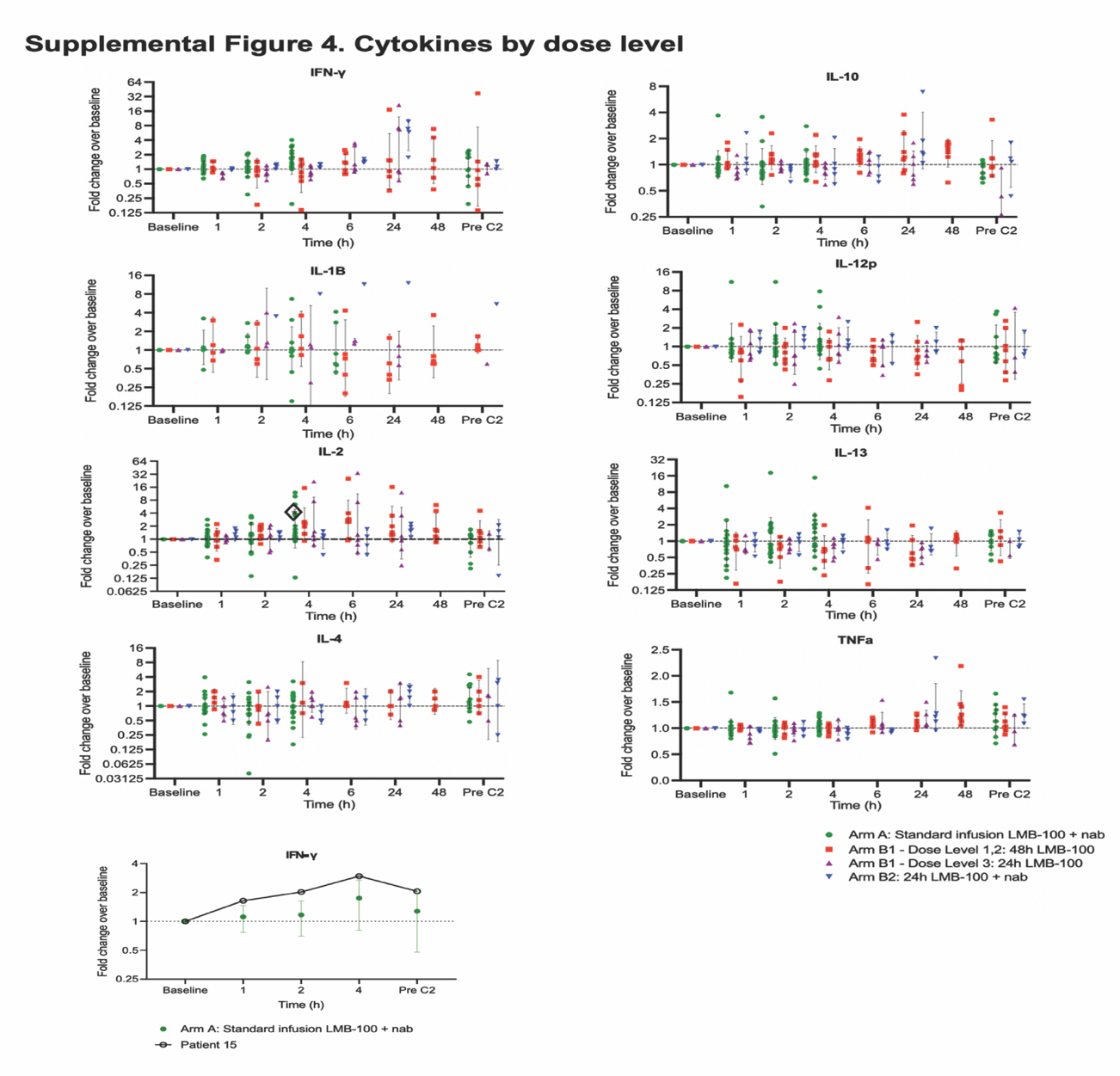

Supplement: Supplementary file 4 — Figure S4 [file CAM4-12-4236-s009.zip › CAM4_5290_Supplemental Figure 4.docx]

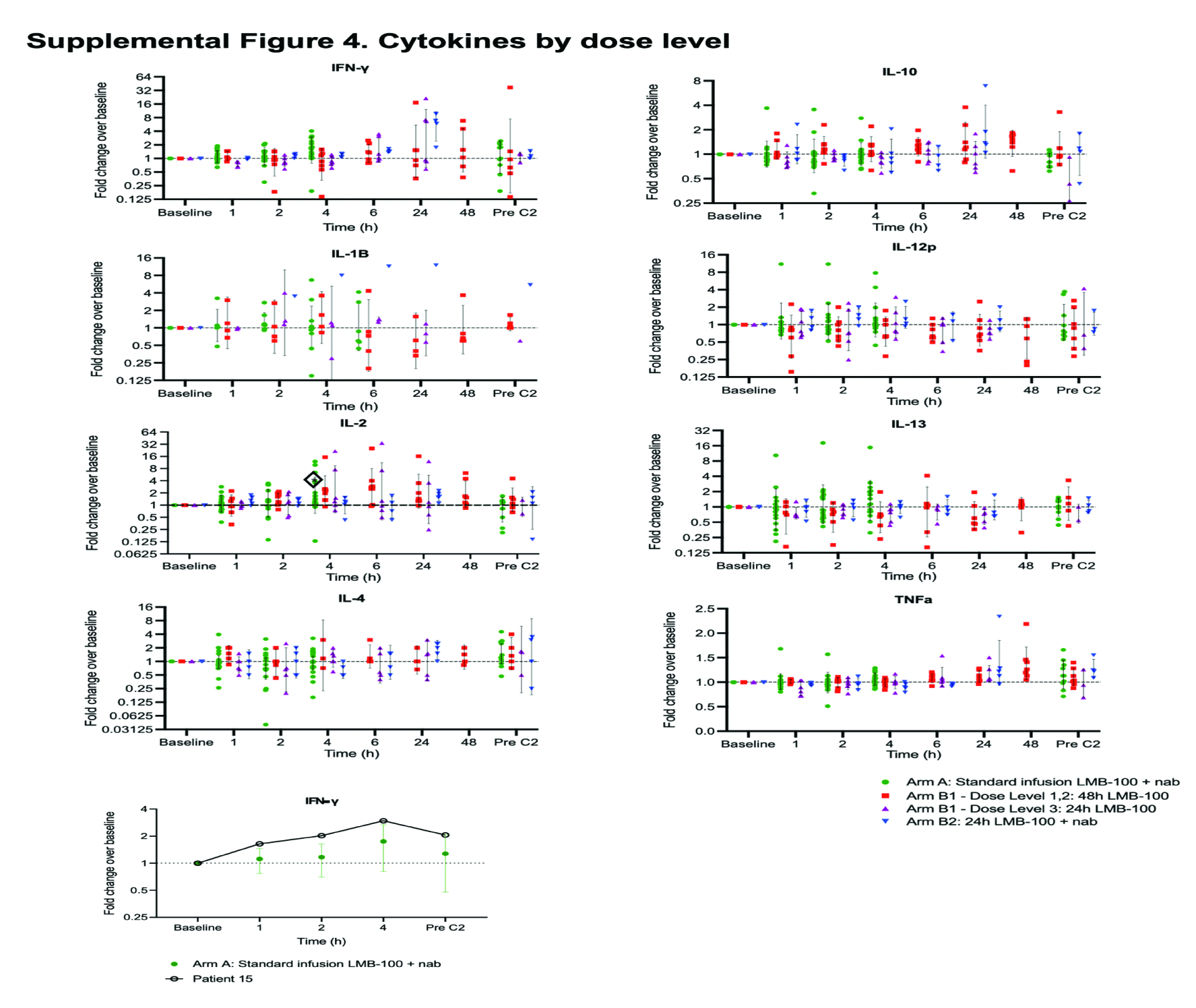

Supplement: Supplementary file 4 — Figure S4 [file CAM4-12-4236-s009.zip › cam45290-sup-0004-FigureS4.tif]

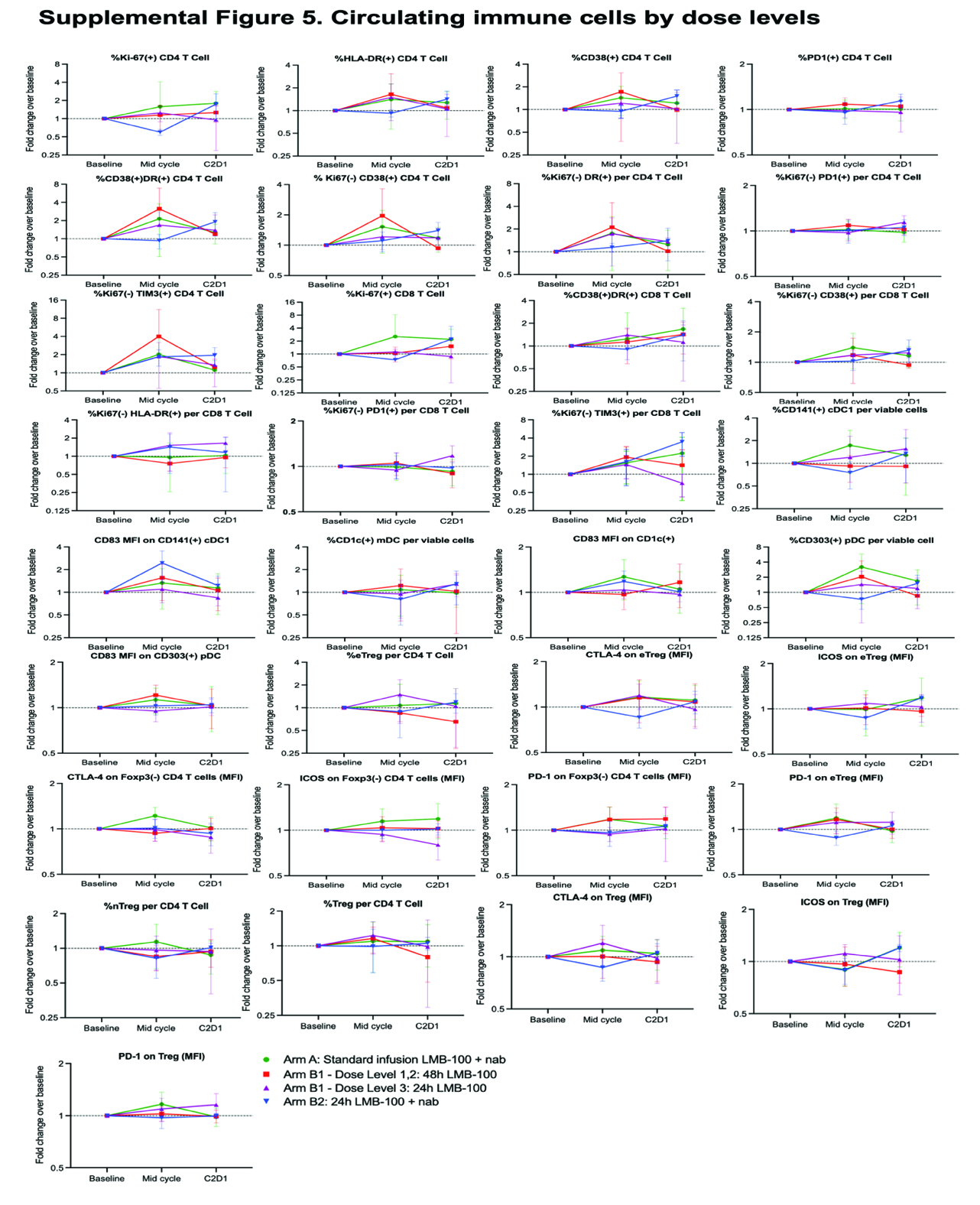

Supplement: Supplementary file 5 — Figure S5 [file CAM4-12-4236-s005.tif]

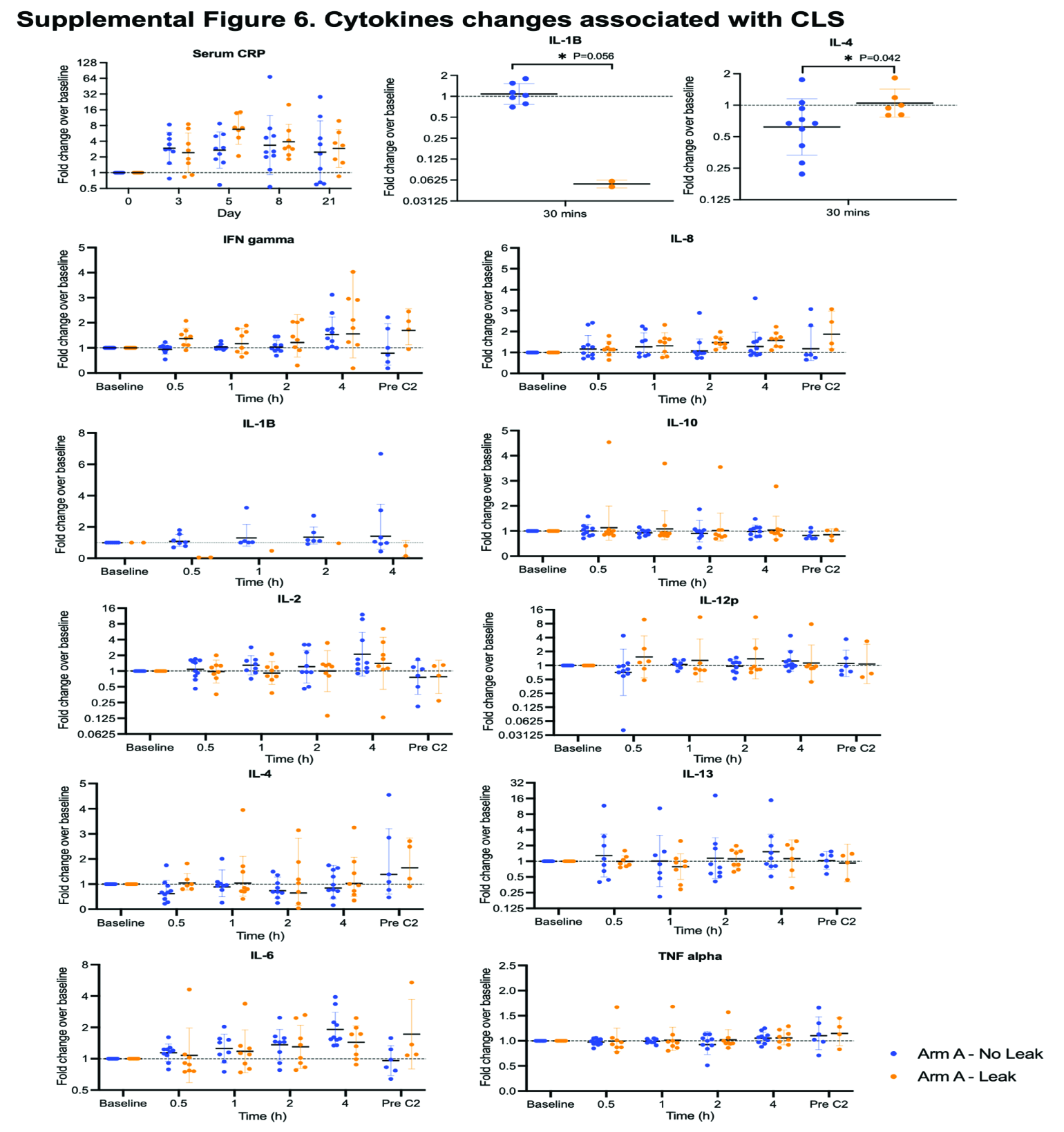

Supplement: Supplementary file 6 — Figure S6 [file CAM4-12-4236-s006.tif]

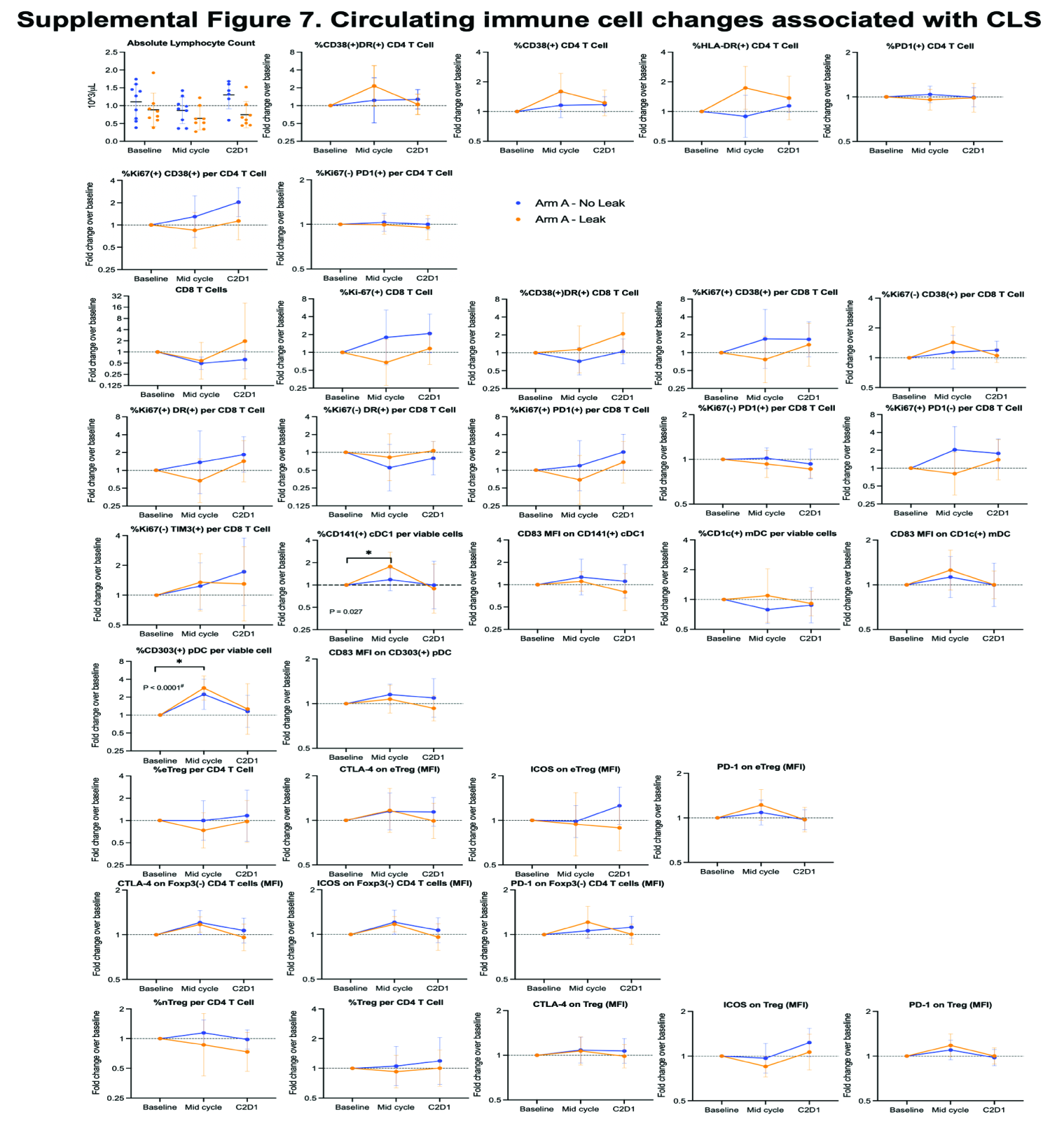

Supplement: Supplementary file 7 — Figure S7 [file CAM4-12-4236-s010.tif]
