# Supplementary material for: Systemic immune changes accompany combination treatment with immunotoxin LMB‐100 and nab‐paclitaxel
Source: Cancer Med. 2022 Oct 8;12(4):4236–49. doi: 10.1002/cam4.5290 (PMC9972172; doi:10.1002/cam4.5290)
Supplement: Supplementary file 8 — Table S1 [file CAM4-12-4236-s001.docx]

| Treatment Unrelated Adverse Events (Grade 3 and 4, all cycles) | | | | | | | | | | | | |  | |
| --- | --- | --- | --- | --- | --- | --- | --- | --- | --- | --- | --- | --- | --- | --- |
|  | Dose Levels and Adverse Event Grade, No. | | | | | | | | | | | |  | |
|  | B1-DL1^a^ (n = 3) | |  | B1-DL2^b^ (n=6) | |  | B1-DL3^c^ (n=6) | |  | B2^d^ (n=5) | |  | |  |
| Adverse Event | Grade >3 | Grade 4 |  | Grade >3 | Grade 4 |  | Grade >3 | Grade 4 |  | Grade >3 | Grade 4 | | | |
| Anemia | 1 |  |  |  |  |  |  |  |  |  |  | | | |
| Hyponatremia | 1 |  |  |  |  |  | 1 |  |  |  |  | | | |
| Alkaline phosphatase increased | 1 |  |  |  |  |  |  |  |  |  |  | | | |
| ALT increased | 2 |  |  |  |  |  |  |  |  |  |  | | | |
| AST increased | 2 |  |  |  |  |  |  |  |  |  |  | | | |
| Hypophosphatemia | 1 |  |  |  |  |  |  |  |  |  |  | | | |
| Lymphocyte count decrease | 1 | 1 |  |  |  |  |  |  |  |  |  | | | |
| Blood bilirubin increase | 1 |  |  |  |  |  | 1 |  |  |  |  | | | |
| Hypoglycemia | 1 | 1 |  |  |  |  |  |  |  |  |  | | | |
| Hyperglycemia | 1 |  |  |  |  |  |  |  |  |  |  | | | |
| Fatigue | 1 |  |  |  |  |  | 2 |  |  |  |  | | | |
| Somnolence | 1 |  |  |  |  |  |  |  |  |  |  | | | |
| Thromboembolic event |  |  |  | 1 | 1 |  |  |  |  |  |  | | | |
| Pleuritic pain |  |  |  |  |  |  | 1 |  |  |  |  | | | |
| Pain |  |  |  |  |  |  | 1 |  |  |  |  | | | |
| Hypotension | 1 |  |  |  |  |  |  |  |  |  |  | | | |
| Edema limbs | 1 |  |  |  |  |  |  |  |  |  |  | | | |
| Sepsis | 1 | 1 |  |  |  |  |  |  |  |  |  | | | |
| Cholangitis | 1 |  |  |  |  |  | 1 |  |  |  |  | | | |
| Liver abscess | 1 |  |  |  |  |  |  |  |  |  |  | | | |
| Nausea |  |  |  | 1 |  |  | 1 |  |  |  |  | | | |
| UTI |  |  |  |  |  |  | 1 |  |  |  |  | | | |
|  |  |  |  |  |  |  |  |  |  |  |  | | | |
| ^a^Arm B1, Dose Level 1: LMB -100 65 mcg/kg 48 hours continuous infusion | | | | | | | | | | | | |  | |
| ^b^Arm B1, Dose Level 2: LMB -100 100 mcg/kg 48 hours continuous infusion with loading dose | | | | | | | | | | | | |  | |
| ^c^Arm B1, Dose Level 3: LMB -100 100 mcg/kg 24 hours continuous infusion with loading dose | | | | | | | | | | | | |  | |
| ^d^Arm B2: LMB -100 100 mcg/kg 24 hours continuous infusion with loading dose and nab-paclitaxel | | | | | | | | | | | | |  | |
