# Supplementary material for: Systemic immune changes accompany combination treatment with immunotoxin LMB‐100 and nab‐paclitaxel
Source: Cancer Med. 2022 Oct 8;12(4):4236–49. doi: 10.1002/cam4.5290 (PMC9972172; doi:10.1002/cam4.5290)
Supplement: Supplementary file 9 — Table S2 [file CAM4-12-4236-s007.zip › CAM4_5290_Supplemental Table 2.pptx]

## Slide 1
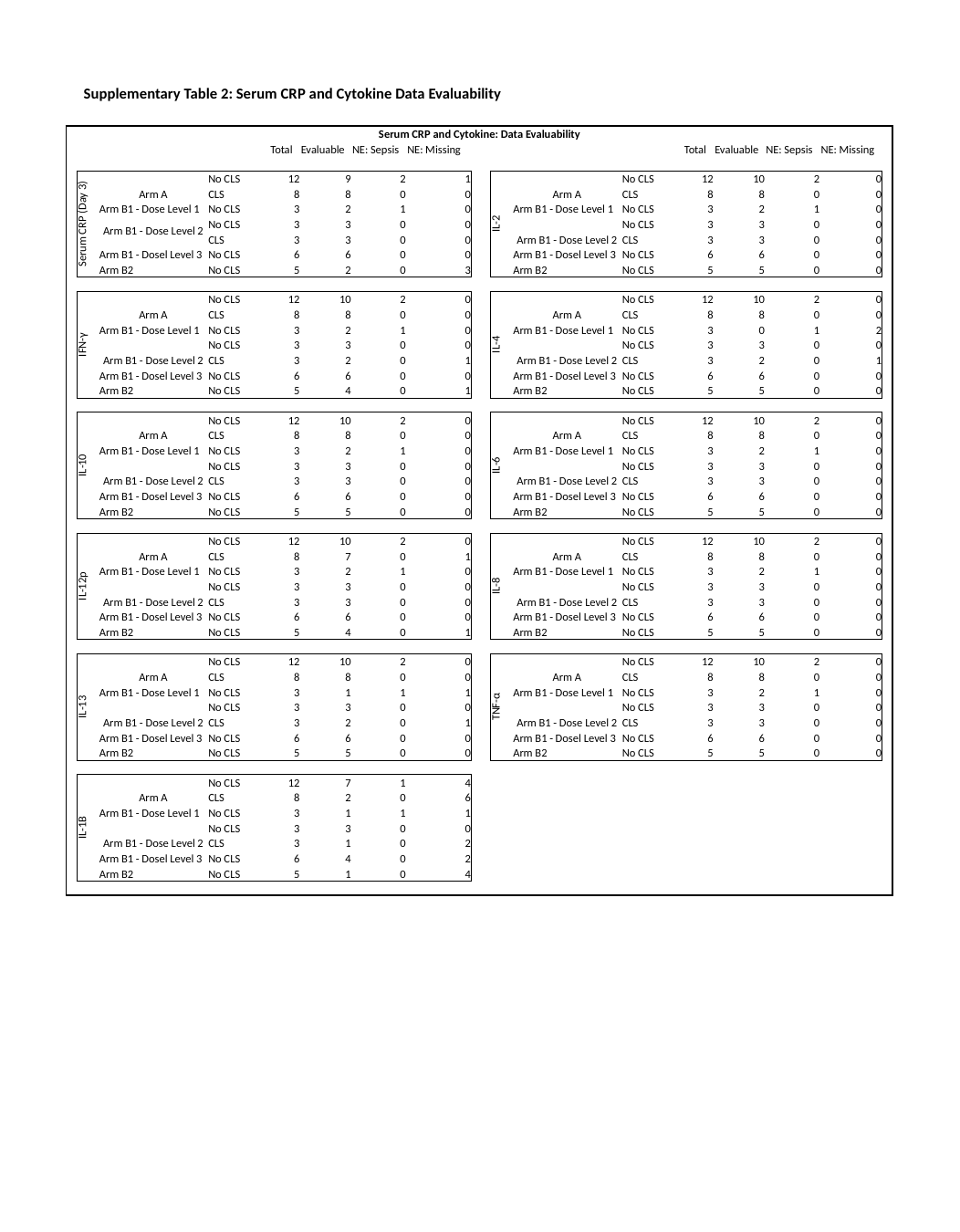

Supplementary Table 2: Serum CRP and Cytokine Data Evaluability
| | Serum CRP and Cytokine: Data Evaluability | | | | | | | | | | | | | | | |
| --- | --- | --- | --- | --- | --- | --- | --- | --- | --- | --- | --- | --- | --- | --- | --- | --- |
| | | | | Total | Evaluable | NE: Sepsis | NE: Missing | | | | | Total | Evaluable | NE: Sepsis | NE: Missing | |
| | | | | | | | | | | | | | | | | |
| | Serum CRP (Day 3) | Arm A | No CLS | 12 | 9 | 2 | 1 | | IL-2 | Arm A | No CLS | 12 | 10 | 2 | 0 | |
| | | | CLS | 8 | 8 | 0 | 0 | | | | CLS | 8 | 8 | 0 | 0 | |
| | | Arm B1 - Dose Level 1 | No CLS | 3 | 2 | 1 | 0 | | | Arm B1 - Dose Level 1 | No CLS | 3 | 2 | 1 | 0 | |
| | | Arm B1 - Dose Level 2 | No CLS | 3 | 3 | 0 | 0 | | | Arm B1 - Dose Level 2 | No CLS | 3 | 3 | 0 | 0 | |
| | | | CLS | 3 | 3 | 0 | 0 | | | | CLS | 3 | 3 | 0 | 0 | |
| | | Arm B1 - Dosel Level 3 | No CLS | 6 | 6 | 0 | 0 | | | Arm B1 - Dosel Level 3 | No CLS | 6 | 6 | 0 | 0 | |
| | | Arm B2 | No CLS | 5 | 2 | 0 | 3 | | | Arm B2 | No CLS | 5 | 5 | 0 | 0 | |
| | | | | | | | | | | | | | | | | |
| | IFN-γ | Arm A | No CLS | 12 | 10 | 2 | 0 | | IL-4 | Arm A | No CLS | 12 | 10 | 2 | 0 | |
| | | | CLS | 8 | 8 | 0 | 0 | | | | CLS | 8 | 8 | 0 | 0 | |
| | | Arm B1 - Dose Level 1 | No CLS | 3 | 2 | 1 | 0 | | | Arm B1 - Dose Level 1 | No CLS | 3 | 0 | 1 | 2 | |
| | | Arm B1 - Dose Level 2 | No CLS | 3 | 3 | 0 | 0 | | | Arm B1 - Dose Level 2 | No CLS | 3 | 3 | 0 | 0 | |
| | | | CLS | 3 | 2 | 0 | 1 | | | | CLS | 3 | 2 | 0 | 1 | |
| | | Arm B1 - Dosel Level 3 | No CLS | 6 | 6 | 0 | 0 | | | Arm B1 - Dosel Level 3 | No CLS | 6 | 6 | 0 | 0 | |
| | | Arm B2 | No CLS | 5 | 4 | 0 | 1 | | | Arm B2 | No CLS | 5 | 5 | 0 | 0 | |
| | | | | | | | | | | | | | | | | |
| | IL-10 | Arm A | No CLS | 12 | 10 | 2 | 0 | | IL-6 | Arm A | No CLS | 12 | 10 | 2 | 0 | |
| | | | CLS | 8 | 8 | 0 | 0 | | | | CLS | 8 | 8 | 0 | 0 | |
| | | Arm B1 - Dose Level 1 | No CLS | 3 | 2 | 1 | 0 | | | Arm B1 - Dose Level 1 | No CLS | 3 | 2 | 1 | 0 | |
| | | Arm B1 - Dose Level 2 | No CLS | 3 | 3 | 0 | 0 | | | Arm B1 - Dose Level 2 | No CLS | 3 | 3 | 0 | 0 | |
| | | | CLS | 3 | 3 | 0 | 0 | | | | CLS | 3 | 3 | 0 | 0 | |
| | | Arm B1 - Dosel Level 3 | No CLS | 6 | 6 | 0 | 0 | | | Arm B1 - Dosel Level 3 | No CLS | 6 | 6 | 0 | 0 | |
| | | Arm B2 | No CLS | 5 | 5 | 0 | 0 | | | Arm B2 | No CLS | 5 | 5 | 0 | 0 | |
| | | | | | | | | | | | | | | | | |
| | IL-12p | Arm A | No CLS | 12 | 10 | 2 | 0 | | IL-8 | Arm A | No CLS | 12 | 10 | 2 | 0 | |
| | | | CLS | 8 | 7 | 0 | 1 | | | | CLS | 8 | 8 | 0 | 0 | |
| | | Arm B1 - Dose Level 1 | No CLS | 3 | 2 | 1 | 0 | | | Arm B1 - Dose Level 1 | No CLS | 3 | 2 | 1 | 0 | |
| | | Arm B1 - Dose Level 2 | No CLS | 3 | 3 | 0 | 0 | | | Arm B1 - Dose Level 2 | No CLS | 3 | 3 | 0 | 0 | |
| | | | CLS | 3 | 3 | 0 | 0 | | | | CLS | 3 | 3 | 0 | 0 | |
| | | Arm B1 - Dosel Level 3 | No CLS | 6 | 6 | 0 | 0 | | | Arm B1 - Dosel Level 3 | No CLS | 6 | 6 | 0 | 0 | |
| | | Arm B2 | No CLS | 5 | 4 | 0 | 1 | | | Arm B2 | No CLS | 5 | 5 | 0 | 0 | |
| | | | | | | | | | | | | | | | | |
| | IL-13 | Arm A | No CLS | 12 | 10 | 2 | 0 | | TNF-α | Arm A | No CLS | 12 | 10 | 2 | 0 | |
| | | | CLS | 8 | 8 | 0 | 0 | | | | CLS | 8 | 8 | 0 | 0 | |
| | | Arm B1 - Dose Level 1 | No CLS | 3 | 1 | 1 | 1 | | | Arm B1 - Dose Level 1 | No CLS | 3 | 2 | 1 | 0 | |
| | | Arm B1 - Dose Level 2 | No CLS | 3 | 3 | 0 | 0 | | | Arm B1 - Dose Level 2 | No CLS | 3 | 3 | 0 | 0 | |
| | | | CLS | 3 | 2 | 0 | 1 | | | | CLS | 3 | 3 | 0 | 0 | |
| | | Arm B1 - Dosel Level 3 | No CLS | 6 | 6 | 0 | 0 | | | Arm B1 - Dosel Level 3 | No CLS | 6 | 6 | 0 | 0 | |
| | | Arm B2 | No CLS | 5 | 5 | 0 | 0 | | | Arm B2 | No CLS | 5 | 5 | 0 | 0 | |
| | | | | | | | | | | | | | | | | |
| | IL-1B | Arm A | No CLS | 12 | 7 | 1 | 4 | | | | | | | | | |
| | | | CLS | 8 | 2 | 0 | 6 | | | | | | | | | |
| | | Arm B1 - Dose Level 1 | No CLS | 3 | 1 | 1 | 1 | | | | | | | | | |
| | | Arm B1 - Dose Level 2 | No CLS | 3 | 3 | 0 | 0 | | | | | | | | | |
| | | | CLS | 3 | 1 | 0 | 2 | | | | | | | | | |
| | | Arm B1 - Dosel Level 3 | No CLS | 6 | 4 | 0 | 2 | | | | | | | | | |
| | | Arm B2 | No CLS | 5 | 1 | 0 | 4 | | | | | | | | | |
| | | | | | | | | | | | | | | | | |
