# Supplementary material for: Systemic immune changes accompany combination treatment with immunotoxin LMB‐100 and nab‐paclitaxel
Source: Cancer Med. 2022 Oct 8;12(4):4236–49. doi: 10.1002/cam4.5290 (PMC9972172; doi:10.1002/cam4.5290)
Supplement: Supplementary file 10 — Appendix S1 [file CAM4-12-4236-s002.docx]

**Supplementary Methods**

**Immune subset analysis**

Multiparameter flow cytometric analysis was performed on PBMCs. Cells were incubated with Fc receptor blocking agent (Miltenyi Biotec) and stained for 20–30 min at 4°C with monoclonal antibodies. For analysis of Foxp3 and Ki67 expression, cells were fixed and permeabilized using a Fix/Perm buffer (eBioscience) according to the manufacturer’s instructions, then stained with anti-Foxp3 antibody. Live cells were discriminated by means of LIVE/DEAD Fixable Aqua Dead Cell Stain (Life Technologies) and dead cells were excluded from all analyses. All flow cytometric analyses were performed using a MACSQuant Analyzer (Miltenyi Biotec). Flow cytometric data were quantified either as the median fluorescence intensity or as a percentage of cells, as indicated. Data were analyzed using FlowJo software version 10.6.1. (FlowJo, LLC).

The following immunophenotypic markers were used to define immune subsets:

CD4+ T cells ; CD8-CD4+

CD8+ T cells ; CD8+CD4+

Tregs ; CD8-CD4+CD25+Foxp3+

eTregs ; CD8-CD4+CD45RA-Foxp3high

nTregs ; CD8-CD4+CD45RA+Foxp3dim

CD1c+ myeloid DC (mDC) ; lineage (CD3, CD19, CD56)−HLA-DR+CD11c+CD1c+

CD141+ mDC ; lineage−HLA–DR+CD11c+CD141+

CD303+ plasmacytoid DC (pDC) ; lineage−HLA–DR+CD11c+CD303+

The following monoclonal antibodies were used:

HLA-DR clone LN3, CD3 clone OKT3, CD56 clone MEM-188, CD19 clone HIB19, CD11c clone Bu15, CD1c clone L161, CD141 clone M80, CD303 clone 201A, CD83 clone HB15e, CD8 clone SK1, CD4 clone RPAT4, CD25 clone BC96, Foxp3 clone 206D, PD-1 clone EH12.2H7, CTLA-4 clone L3D10, TIM-3 clone F38-2E2 ICOS clone C398.4A, CD45RA clone HI100, CD38 clone HIT2 (BioLegend) and Ki67 clone B56 (BD Biosciences).
